# A miR396d-PagGRF20-PagXTH5 module balances growth and salt tolerance in poplar

**DOI:** 10.1093/plphys/kiag328

**Published:** 2026-06-02

**Authors:** Avilash Singh Yadav

**Affiliations:** Assistant Features Editor, Plant Physiology, American Society of Plant Biology, United States; Weill Institute for Cell and Molecular Biology and Section of Plant Biology, School of Integrative Plant Sciences, Cornell University, Ithaca, NY 14853, United States

Salinity is a major limitation for agricultural productivity and stability of forest ecosystems (Tarolli et al. 2024). Excess salt buildup near the root zone lowers the soil water potential, which limits water uptake by plants. The resulting reduction in water availability induces osmotic stress, disrupts ion homeostasis, and promotes reactive oxygen species (ROS) production, each contributing to plant growth inhibition (Fu and Yang 2023). Perennial woody plants are particularly vulnerable to salinity as they gradually accumulate toxic levels of ions, causing severe physiological damage (Lu et al. 2021). Salinity can make the plants susceptible to dehydration by impairing xylem hydraulic conductivity (de Jesus et al. 2025). Salinity can also alter cell wall composition and disrupt the balance between wall extensibility needed for growth and wall rigidity required for maintaining structural integrity (Colin et al. 2023).

Plants use multiple adaptive strategies, such as restricting ion uptake and limiting oxidative damage, to cope with salinity stress (Balasubramaniam et al. 2023). Although the physiological responses to salinity stress have been extensively studied, how plants regulate cell wall properties to impart salt tolerance remain poorly understood.

Cell wall modifying enzymes, such as xyloglucan endotransglucosylase/hydrolases (XTHs) that modify xyloglucan chains, contribute to osmotic stress tolerance by remodeling the xyloglucan-cellulose network of the cell walls (Eklöf and Brumer 2010; Jiang et al. 2020). Although cell wall loosening promotes growth, wall reinforcement is also important to withstand stress conditions (Colin et al. 2023). Therefore, the extent of wall remodeling needs to be tightly regulated. Precisely how the expression and activity of XTHs are regulated under salinity conditions to balance wall stiffness and wall extensibility remain unclear.

Gene expression is tightly regulated by transcription factors and post-transcriptional modifiers such as microRNAs (miRNAs). These miRNAs are small (20–24 nt) non-coding RNAs that bind to target transcripts via sequence complementarity, resulting in target mRNA cleavage and translational repression (Borges and Martienssen 2015). Among them, the miR396 family of miRNAs target GROWTH REGULATING FACTOR (GRF) transcription factors. While GRFs are known to promote cell proliferation and growth, emerging evidence shows that GRFs also mediate adaptation to salt or drought stress (Omidbakhshfard et al. 2015). In woody plants such as poplar, the PagGRF20-PagMYB4 module was shown to promote wood formation, although how PagGRF20 modulates cell wall dynamics was unknown (Wang et al. 2026a, 2026b). Whether PagGRF20 contributes to salt stress tolerance, and whether this process occurs via XTH-mediated cell wall remodeling remains unclear.

In this issue of *Plant Physiology*, Wang et al (Wang et al. 2026a) demonstrated how PagGRF20 negatively regulates salt tolerance in poplar. The authors initially constructed a gene co-expression network and identified PagGRF20 as a key regulator that was associated with genes involved in cell wall integrity and stress responses. RNA-seq and RT-qPCR analyses further showed that *PagGRF20* expression was strongly attenuated under salt stress. To test the role of PagGRF20 in salinity response, the authors generated *PagGRF20*-overexpression (*PagGRF20*-OE), *PagGRF20*-RNA interference (*PagGRF20-*RNAi) and *paggrf20* CRISPR/Cas9 knockout lines. Under normal conditions, *PagGRF20*-OE lines developed smaller leaves, whereas both *paggrf20* knockout and RNAi lines exhibited increased leaf size. However, *PagGRF20*-OE plants exhibited increased wilting and elevated ROS levels under salt stress conditions. In contrast, the *paggrf20* knockout and RNAi lines showed only mild drooping and improved water retention, along with reduced ROS levels, suggesting that *PagGRF20* negatively regulates salt tolerance.

To determine how PagGRF20 regulates salt sensitivity, the authors compared the chromatin accessibility profile between *PagGRF20*-OE and the wild-type (WT) plants. Regions with increased accessibility in *PagGRF20*-OE lines were associated with growth related pathways, whereas those with reduced accessibility were enriched in stress responsive genes. To identify direct targets of PagGRF20, the authors integrated chromatin accessibility, DNA-binding profiles, and transcriptomic datasets, revealing that PagGRF20 is a key regulator of genes involved in cell wall biogenesis and remodeling pathways. Among these genes, PAgGRF20 was found to directly bind to the promoter of *PagXTH5* to activate *PagXTH5* expression (Fig. 1), and this finding was validated using multiple independent assays. Finally, analysis of cell wall composition under salt stress conditions showed that while the WT plants showed increased lignification to reinforce the cell walls, the *PagGRF20*-OE lines failed to accumulate similar levels of lignin. Overall, these findings are consistent with the idea that plant salt tolerance requires a balance between wall loosening and stiffness, where PAgGRF20 directly regulates *PagXTH5* to coordinate this balance and modulate salinity tolerance.

**Figure 1:**
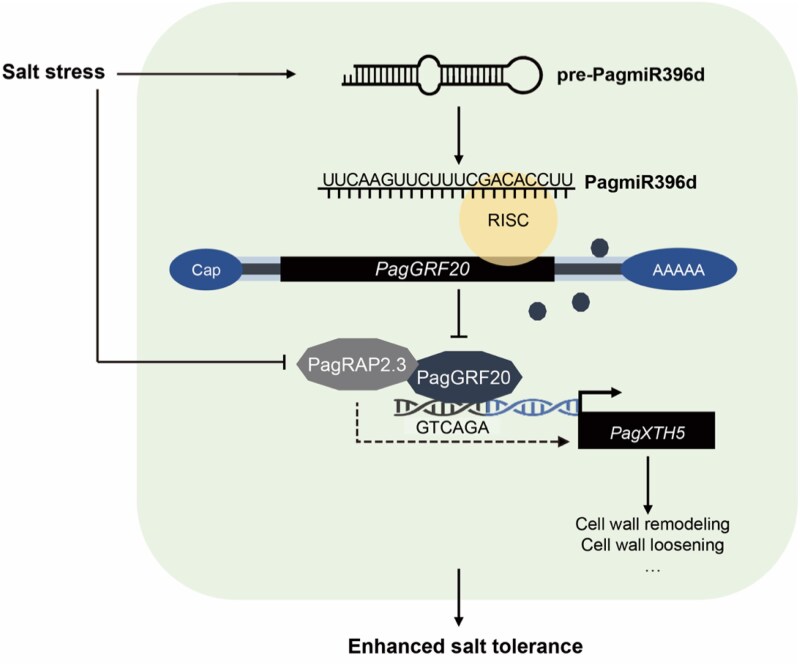
Schematic shows how the mir396d-PagGRF20-PagXTH5 module confers salinity tolerance in poplar. Under normal conditions, PagGRF20 directly binds to the GTCAGA motif in the *PagXTH5* promoter to activate *PagXTH5* expression. PagRAP2.3 functions as a co-activator, wherein PagRAP2.3 physically interacts with PagGRF20 to enhance the activation of *PagXTH5*. Since PagXTH5 exhibits XET activity, elevated PagXTH5 results in XET-mediated cell wall remodeling and wall loosening to promote growth. However, salt stress conditions promote the accumulation of miR396d, which in turn represses *PagGRF20* transcripts via RNA-induced silencing complex (RISC)-mediated cleavage. Reduced levels of PagGRF20 protein subsequently compromises *PagXTH5* activation. Consequently, reduced PagXTH5 activity attenuates cell wall remodeling to promote cell wall reinforcement and maintenance of structural integrity, thereby enhancing salt tolerance in poplar. Reproduced from (Wang et al. 2026a).

How might PagXTH5 contribute to salt tolerance? Overexpression of *PagXTH5* (PagXTH5-OE) showed reduced tolerance under salt stress conditions, characterized by severe wilting, dehydration, and increased levels of ROS. Sequence analysis suggested that PagXTH5 may function as a xyloglucan endotransglycosylase (XET), where XET activity modifies cell wall properties by cleaving and rejoining xyloglucan chains. The authors found that the *PagXTH5*-OE lines indeed showed increased XET protein abundance. Moreover, *PagGRF20*-OE lines also showed increased XET abundance, whereas XET levels were reduced in *paggrf20*/RNAi lines, indicating that PagGRF20 promotes XET accumulation through PagXTH5. Elevated XET activity in *PagXTH5*-OE lines also resulted in the hyperaccumulation of cellulose and hemicellulose, together with reduced lignin content relative to the WT. In summary, these findings show that PagXTH5, which is directly regulated by PagGRF20, promotes cell wall remodeling while compromising salt tolerance in poplar.

The authors also identified an ethylene-responsive transcriptional activator PagRAP2.3 that physically interacts with PagGRF20. Based on in vitro GST (glutathione S-transferase) pull-down and in vivo bimolecular fluorescence complementation assays, the authors confirmed that PagRAP2.3 physically interacts with PagGRF20 in the nucleus. Although PagRAP2.3 did not bind directly to *PagXTH5* promoter, EMSA (electrophoretic mobility shift assays) and effector-reporter based gene transactivation assays showed that PagRAP2.3 may function as a transcriptional co-activator to enhance PagGRF20 mediated activation of PagXTH5.

Since GRFs are regulated by miR396, Wang et al also investigated how miR396d regulates PagGRF20 to confer salt tolerance. As expected, salt stress led to the upregulation of *miR396* and corresponding downregulation of *PagGRF20* and *PagXTH5* (Fig. 1). Dual-luciferase assays confirmed that miR396d directly represses *PagGRF20* transcripts. Moreover, *PagGRF20* expression was found to be consistently lower in *35S:miR396d* lines under both normal and salt stress conditions. The *35S:miR396d* lines also showed reduced wilting of leaves, increased water retention, lower ROS accumulation, and increased activity of ROS scavenging enzymes compared to WT.

In summary, Wang, Guan and colleagues (Wang et al. 2026a) demonstrated that under salt stress conditions, miR396d suppresses PagGRF20 to limit PagXTH5-mediated cell wall remodeling and confer salt tolerance in poplar. This study addresses an important knowledge gap in how a model perennial, poplar, coordinates the trade-off between cell wall remodeling mediated growth and resilience to salinity stress. Breeding strategies in the future can target the miR396d-PagGRF20-PagXTH5 module to improve salt tolerance in woody plants, although the effects on growth and wall integrity need to be optimized carefully.

## Data Availability

There is no new data associated with this News & Views article.
